# Medical Bristol in the Eighteenth Century

**Published:** 1899-12

**Authors:** W. H. Harsant

**Affiliations:** Surgeon to the Bristol Royal Infirmary.


The Bristol

Medico-Chirurgical Journal.

DECEMBER, 1899.

### MEDICAL BRISTOL IN THE EIGHTEENTH CENTURY.

The Presidential Address, delivered on October 11th, 1899, at the Opening of the Twenty-sixth Session of the Bristol Medico-Chirurgical Society.

BY

W. H. Harsant, F.R.C.S. Eng.,

*Surgeon to the Bristol Royal Infirmary.*

Although I have been a member of this Society for twenty-three years, and during that time have been actively engaged in the practice of surgery, I am conscious that such a period is not long enough to justify me in inflicting upon you anything in the nature of reminiscences, or of comparisons between the state of our knowledge at the commencement of my career and our more enlightened condition at the present time.

I cannot, like some of my predecessors in this chair, go back to the pre-anæsthetic period and draw attention to the wonderful advancement made in surgery since that time, and although I might tell much of the pre-antiseptic days and recall many of the glorious improvements which have been effected by the introduction of the antiseptic and aseptic treatment of wounds, it seems to me that such a subject is somewhat hackneyed; it has been done so many times before by abler hands than mine, that I prefer to address you to-night in a lighter vein, and if possible to interest you for a short time in some of the doings and sayings of our predecessors who fretted their time upon the stage in Bristol before any of us now present had arrived upon the scene.

I have lately had the opportunity of looking through some old manuscript volumes of "Biographical Memoirs," written by Richard Smith, a surgeon who lived in Bristol at the beginning of the present century, and I thought that inasmuch as these volumes, fifteen in number, were never published, and are inaccessible to many of the members of this Society, some account of their contents might be of interest. I therefore purpose to dive into their pages and to extract some of the interesting gossip which they contain about the medical profession in Bristol during the latter half of the eighteenth century. And first I would say a few words about the writer of the volumes. He says little about himself. Although he took infinite pains to gather particulars of his contemporaries and predecessors, we are left to other sources to find out what we know concerning the author.

He was the son of a former Richard Smith, surgeon to the Infirmary from 1774 to 1791. He himself was surgeon to that institution from 1796 to 1843, a period of no less than forty-seven years, so that father and son continued in office over an almost uninterrupted period of seventy years.

The elder Richard Smith lived at first in Charlotte Street at the corner of Queen Square; he afterwards moved to a house in College Green near St. Augustine's Church, on the site of the present Royal Hotel, where he practised until he died in 1796. He seems to have been a man of great ability and industry, and bequeathed to his son a museum containing nearly a thousand specimens, which were afterwards presented by the son to the Infirmary, forming the nucleus of what is now called Richard Smith's Museum.

The younger Richard Smith resembled his father in many respects, especially in his love of collecting specimens. He was particularly partial to monstrosities and freaks, and such objects as the skeleton of a murderer or the enormous penis of a negro were to him veritable gems, to be obtained and preserved at all costs.

Mr. Augustin Prichard in his *Reminiscences* describes Richard Smith as he remembered him. He was then senior surgeon to the Infirmary, and very old and thoroughly incapable; so much so, that when he died a law limiting the tenure of office to twenty years was passed. He was universally known as "Dick Smith," and, wrapped in a rough camlet cloak, used to drive about in a gig, with a white dog running underneath. He was a Freemason, very high up in the craft, and was a *beau idéal* chairman at the jovial suppers the brethren were in the habit of giving.

He commenced practice in 1795, and lived at first in 17 College Street, in a house having a back entrance in Lamb Street. In 1803 he went to live at 7 College Green, where he remained until he died in 1843. His death took place very suddenly while he was sitting in a chair in the committee-room of the Philosophical Institution. He was one of the original members of the Medical Reading Society, founded in 1804, which still exists in a flourishing state, and is now approaching its centenary.

He seems to have taken a morbid delight in adding to his museum any specimens relating to murder trials, and no effort was spared on his part to bring the unfortunate prisoner safely to the gallows, and afterwards to secure the body for dissection and the skeleton for the museum. Full particulars of several such trials have been preserved by him in manuscript volumes, which contain many curious details and illustrate in a ghastly manner the craze which then existed for capital punishment.

Among his collection is the articulated skeleton of John Horwood, a man who was hanged in 1821 at the gaol on the Cut for the murder of his sweetheart. Many of my hearers who have been students at the Infirmary are familiar with the old volume in the Museum, bound in human skin, so carefully preserved by Richard Smith, and containing such a detailed account of the trial and execution of this man.

The body of the murderer having been ordered, as a part of the sentence, to be given over to the surgeons of the Infirmary for dissection, Richard Smith went himself in a coach to the gaol and brought away the body to the Infirmary, wrapped up in an old cloak. It was actually placed on the table in the operation room and allowed to remain there for four days, while Richard Smith gave popular anatomical demonstrations to any citizens of Bristol who chose to attend. No less than eighty persons were attracted on the first day alone to this gruesome spectacle, and on the succeeding days there were also very full audiences, while finally the students of the house were allowed to finish the dissection of the body in the dissecting-room which then adjoined the dead-house. The skeleton was afterwards articulated and now hangs in the Infirmary museum, while the skin underwent a process of tanning and now serves as a binding for the volume which contains a record of the proceedings.

This handing of the bodies of murderers to the surgeons of the Infirmary for dissection seems to have been a long-established custom in Bristol, for in 1741, when Captain Goodere was executed for the murder of his brother on the high seas, his body was removed to the Infirmary, where it was placed in the operation room, and, in the presence of as many spectators as the room would hold, a surgeon stuck a scalpel into the breast. In this state it was exposed to the popular gaze until the evening, and then given over to the friends for burial.

Another instance of the same kind occurred in 1802, when two young women, named Charlotte Bobbett and Maria Davis, were hanged for infanticide. Their bodies were given over to the surgeons of the Infirmary for dissection, and after execution at St. Michael's Hill gallows they were conveyed to the Infirmary in an open cart, followed by an immense mob. The surgeons were in attendance to receive them, and after the bodies had been stripped and laid out on the table of the committee room a crucial incision was made in the chest of each by Mr. Godfrey Lowe, the senior surgeon, in the presence of as many of the rabble as were able to crush into the room.

On the following day, at the request of the Mayor and Aldermen, who were present, the brain of one of the girls was dissected and lectured on by Richard Smith, then the junior surgeon. The articulated skeletons of these young women now hang in the Infirmary museum, and particulars of the trial are fully recorded in a separate manuscript volume.

### CLASSES OF PRACTITIONERS.

The members of the profession last century were divided into four classes; viz., physicians, surgeons, barber-surgeons, and apothecaries. In 1754 there were in Bristol 5 physicians, 19 surgeons, 13 barber-surgeons, and 29 apothecaries, making a total of 66. In 1793 there were no more barber-surgeons, but the number of apothecaries had risen from 29 to 35, and the surgeons from 19 to 20, while the number of physicians seems to have remained about the same as before.

### PHYSICIANS.

The physicians held their heads very high. They were some of the best educated men of that day. They all had a good knowledge of the classics, and many of them went to foreign universities for a part of their professional training. They were very particular in their dress and deportment, and were easily to be distinguished from the common herd. Thus of Dr. Wm. Logan, one of the first physicians to the Infirmary, we read: "He was a strict observer of professional costume, and never stirred abroad or was visible at home, unless in full dress, that is to say, his head covered by the immense flowing wig of George II.'s time, a red roquelaire hanging from his shoulders to his heels, his wrist graced by a gold-headed cane, and his side furnished with a long French rapier."

Of Dr. Ludlow, physician to the Infirmary in 1774, we read: "He was distinguished from the common mass by an imposing exterior. He moved in a measured step and affected a meditative abstraction of countenance, with a pomposity of diction and manner which could not but keep the vulgar at a respectable distance. His peruke alone was enough in itself to command respect, for it was ornamented with regular and formidable tiers of curls to the number of one hundred and ten, and was known as 'The Royal George,' in allusion to the great line-of-battle ship, then the pride of the navy."

So also of Dr. John Wright, physician in 1771, we read: "His practice was extensive and laborious, so that latterly he was obliged to keep a horse, which was rather unusual for an M.D. in his day. An observer told Richard Smith that he was passing Stoke's Croft one very wet day when Dr. Wright turned the corner; he had on a large white dishevelled wig, over which hung a huge flapped Quaker's hat. With one hand he held up an umbrella, and with the other the bridle of a little pony upon which he rode; from his shoulders his red roquelaire was spread over the hind quarters of the animal, reaching almost to the ground, and forming altogether a caricature so droll that everybody turned round to look at him."

It was customary for the surgeons of the Infirmary also to wear a wig and the red cloak known as the roquelaire, also to carry a gold-headed cane. This fashion prevailed for about forty years after the foundation of the charity in 1735; but when Mr. Noble was elected in 1776, he refused to comply with the custom, in consequence of which the other surgeons agreed to wear out the cloaks in their possession and then to allow the custom to drop. Few of the faculty of the city wore them when Richard Smith first remembered the house in 1787, but Dr. Plomer wore his until 1795, and Dr. Wright until 1798.

Dr. Middleton, who was elected to the Infirmary in 1737, was the first physician who kept his carriage in Bristol. It was a great lumbering thing without springs, with two small glasses in the doors. The horses never went beyond a foot pace, in fact it was a sort of genteel waggon.

Physicians were called in chiefly when the sick person was *in extremis;* in fact, as they then complained, "they came only to administer musk and to close the eyes of the patient!" The practice of giving musk began to decline about the year 1790. Richard Smith could remember when it might be smelled in the street as you passed the house of a dying person, as very few who could afford to pay for it were allowed to depart without having it administered.

When Dr. John Paul was physician to the Infirmary, from 1772 to 1775, the surgeons of the day called him "their good friend Sangrado," since the minute he was in attendance one of them was sent for to make use of his lancet. Mr. Metford used to say that he had bled thirty patients a day at the Infirmary by Dr. Paul's order, and that he was occasionally in the admission-room when Dr. Paul was taking in, and that the first question he asked of every male patient was, "Are you a Bristol man?" If the reply was in the affirmative, he regularly wrote in his book "V. S. ad ℥xx" by way of beginning. Mr. Metford requested to know why, without further enquiry into their complaints, he ordered them to lose so much blood. "Because, sir," said Dr. Paul, "if he is a Bristol man, I know that he sits of an evening smoking tobacco and drinking your abominable fat ale; the first thing to be done therefore is to let some of that run out, and then we shall see what else is the matter."

### SURGEONS.

The surgeons seem to have been largely occupied in bleeding patients at the request of the physicians, but they performed all the operations that were done at that day, the chie of which was to cut for the stone. In an old newspaper, called the *Bristol Weekly Intelligencer*, we find the following paragraphs:—

1742. "Last week two boys were cut for the stone by Mr. Thornhill, both of which are in a fair way of recovery."

May, 1750. "Within these few weeks past four boys have been cut for the stone in the Bristol Infirmary by Mr. Thornhill, and are all recovered. We hear he has more patients preparing for the same operation."

And in January, 1751. "Last week the operation for the double hare-lip was performed at the Bristol Infirmary. At the same charity, a man, aged 70, and two boys were cut for the stone, all which have had the most favourable symptoms and are in a fair way of recovery."

We see that surgeons understood the art of advertising even in those days, and it is not left to these degenerate times to discover a method of bringing an operator's name before the admiring gaze of the public.

The Wm. Thornhill alluded to above was one of the original surgeons of the Infirmary. Richard Smith describes him as a man of much genius and ability, but wanting in attention and assiduity—in fact, he was so remiss in his attendance that he more than once fell under the censure of the house visitors, and finally an incident occurred which I will relate, as it is told by Richard Smith, inasmuch as it exemplifies the way they then looked upon questions of payment in the matter of hospitals.

In 1754 a boy had been wounded in the leg by a gentleman who was shooting, and was brought as a patient to the Infirmary, where he was under the care of Mr. Thornhill, who seems to have neglected to see him. The gentleman, being very anxious respecting the lad, called at Thornhill's house, begged that he would see the boy, and by way of quickening his attention gave him a fee, which the surgeon put in his pocket. This coming to the ear of the house visitors, they considered it their duty to notice it in an official manner, first as an instance of dereliction of duty, and next as militating against one of the first and fundamental rules of the charity whose great feature is gratuitous assistance. Mr. Thornhill's friends advised an immediate resignation in order to prevent the intended report of the transaction, but he clung to the office, promised and retracted, heaping delay upon delay, in the hope that he might soften his accusers; but they were inflexible. At last he gave notice that he would resign in eight months. While this proposition was before the house visitors the secret leaked out, the city was soon in motion, others canvassed for the expected vacancy, and at last the nuisance became so intolerable that the subscribers proceeded to an election, and forced him from his seat in November, 1754. Mr. Thornhill nevertheless refused to consider that his functions were at an end, and the subscribers saw with astonishment six surgeons jostling each other and quarrelling about the proprietorship of the unfortunate patients. The fact was, Thornhill was determined not to be turned out, and therefore held the situation until June 2nd, 1755, the time which he had chalked out for himself; then he bade good-bye to the charity, the committee of which had the politeness afterwards to depute Dr. Bonython and Mr. Page to wait on him with "the thanks of their meeting for the care he had taken of the house from the beginning, and for his many services to it." When in practice he resided for some time in a large corner house, turning from the end of College Green to St. Augustine's Back, and afterwards in a house next St. Werburgh's Church, now the site of the Commercial Rooms. He was a handsome, well-grown man, and took care to show his person to advantage by constantly wearing an entire suit of black velvet and an elegant steel-handled rapier. He was well read, and polished in his manner. He kept a well-appointed equipage, an almost unprecedented luxury in those days, and lived altogether in a much better style than any surgeon in Bristol for many years after him or before him.

Richard Smith gives us a description of another surgeon to the Infirmary, Mr. John Townsend, who held office for twenty-five years from 1755 to 1780. His entire devotion to business procured him so much practice that in 1778 he set up his carriage, a matter of some consequence in those days, there having been only two surgeons before him who did so; viz., his master, Mr. Thornhill, and Mr. Peter Wells. He entered into contract with one Thomas Jones, who agreed to find him a day coachman and a night coachman, a vehicle and a pair of horses, for £100 per annum—a price which may well make us envious in these days, although we must remember that £100 then was probably equal to double that sum now. His drivers had no sinecure places, being in use almost perpetually. He resided in Broad Street, having a side door inside a house passage; this was then considered a great desideratum, as all venereal patients were in the habit of sneaking into a surgery after dusk, privately, and the greatest care was taken to conceal them. Mr. Townsend's surgery was in itself calculated to strike terror into all beholders. It was fitted up with glass cases, and there were exhibited in great display an iron screw ambé, for the reduction of dislocated shoulders; all the endless

"apparatus major" for cutting for the stone, with an endless variety of blunt gorgets and dilating forceps, old actual cauteries, and in fact all the farriery of Scultetus, Hildanus, and Ambroise Paré. One might easily have fancied oneself in the torture room of a Spanish Inquisition, and surrounded with the instruments of "The Question." Mr. Townsend prided himself not a little upon the possession and display of these chirurgical treasures, for he considered, and it was so then to a certain extent, that the minds of the vulgar measured by them the abilities of the possessor. Chatterton, whom nothing of this sort escaped, used to come there occasionally to see Richard Smith, who was indentured to Mr. Townsend, and when he afterwards introduced them both into a satire, speaks of Townsend as—

> "A thing of flatulence and noise,
> Whose surgery's nothing but a heap of toys."

Nothing could be more uncouth than Townsend's manner towards his patients, public and private, for he made no distinction. He overawed them all by his taciturnity and the sternness of his brow. In his person and appearance he greatly resembled Dr. Johnson. His costume never varied; he wore a large unpowdered wig, with a cocked hat, an entire suit of dark snuff-coloured cloth, worsted stockings, square-topped shoes, and small silver knee and shoe buckles; his waistcoat had two large flaps hanging half-way down his thighs, and in his coat he had always four pockets, generally filled with a tow bag and instrument cases. He was so great an economist of time that he had fitted up in the front of his carriage a spreading board with tin cases for ointments, a spatula, and a drawer for white and brown tow. He could be seen, as he rode along, engaged in pulling and spreading of pledgets.

He was once walking down Broad Street during an illumination, and observed a boy breaking every window which had not a light. He asked him how he dared to injure people's property in that way. "Oh," said the boy, "all for the good of trade; I am a glazier!" "All for the good of trade is it?"

said Townsend, lifting up his cane and breaking the boy's head: "there then, you rascal, get that mended for the good of my trade; I am a surgeon."

### BARBER-SURGEONS.

It is well known that at one time surgeons were united with barbers, and belonged to the United Worshipful Society of Barber Surgeons. This Society had for its Hall in Bristol, during last century, a large room in the West India Coffee House, in the Market Place, near the Exchange. This room was known as "The Surgeons' Hall," and it was here that the first meeting of the governors of the Infirmary was held, in 1737, to elect their first Faculty. In the year 1745 the surgeons were emancipated from the barbers, and were obliged to leave all their joint stock in the hands of the latter, and this Hall among the rest.

Mr. Thornhill served his time to a barber-surgeon, known as "Old Rosewell," who, to the day of his death, had at his door in All Saints' Lane the insignia of his trade; viz., a staff, a porringer, and a red garter. This mode of education was then so universal and regular, that in a controversy which took place in 1754 it was asserted as a recommendation to Mr. James Ford "that he was regularly educated in this city in a barber's shop."

When being bled, a patient would grasp a staff or pole, which the barber-surgeon always kept handy. To this staff was tied the tape used in bandaging the patient's arm. When not in use, the pole was hung outside as a sign of the trade followed within. Later, the identical pole used by the customers was not exhibited as a sign, but, instead, a painted pole was placed at the doorway. At first barber-surgeons' poles were painted red and white, while those of mere barbers were required to be white and blue. This law was enforced in England up to 1729.

The newspapers of the day designate Mr. Rosewell as "an eminent surgeon, with a fair character." Thornhill learned here, after the custom of the day, to shave, bleed, and draw teeth; his master being so celebrated that on a Sunday morning there were swarms of persons to be bled, for which each paid from sixpence to a shilling.

An advertisement in *Felix Farley's Journal* for March 9th, 1754, reads: "Henry Haines, barber, Redcliff Pit, shaves each person for two pence, cuts hair for three half-pence, and bleeds for sixpence. All customers who are bled he treats with two quarts of good ale, and those whom he shaves or cuts their hair with a pint each."

The last remnant of barber-surgery dropped with "Old Parsley," who lived next door to the Guildhall in Broad Street as late as the year 1807. This man dressed more wigs, drew more teeth, and spilled more blood than any man in Bristol. At his window and by the side of his door hung immense double strings of teeth. He regularly brought his patients to the door, either for the sake of a good light or for notoriety.

### APOTHECARIES.

Most of the general practice of the city was then done by the apothecaries, who were a most important body of men. They dispensed their own medicines, and many of them, in addition, kept open shops for the sale of drugs. At the foundation of the Infirmary a resident apothecary was appointed to do the dispensing and to live on the premises, and inasmuch as he was the only resident officer he must have acted in addition as a kind of house surgeon. He was an unmarried man, and he had the magnificent salary of £30 per annum; but it seems to have been an appointment much sought after, and it evidently was a position of considerable importance. At many of the large London hospitals, the office of apothecary continued until quite recently, and even in my student days at Guy's Hospital Mr. James Stocker filled this office, the duties of which were very varied and dignified.

### LECTURES.

There was no medical school in Bristol until 1833, but lectures on various subjects, such as Anatomy, Physiology, and Surgery, were given from time to time by members of the Infirmary staff and others, and full particulars of these courses of lectures may be found in Mr. John Latimer's *Annals*, chiefly drawn from Richard Smith's "Memoirs."

Mr. Frank Bowles gave anatomical instruction to students of the Infirmary gratuitously. This was before he became surgeon to the institution, and the work was carried on under great difficulties. The principal difficulty then, as now, was to obtain subjects for dissection. The students (Richard Smith among the foremost) played the part of resurrection men, and procured subjects in succession. In doing this they more than once got themselves into awkward scrapes, and one night Robert Lax and Richard Smith were actually shot at while engaged in abstracting a body from the Infirmary burial ground in Johnny Ball Lane.

They used to substitute old sacks filled with rubbish for the contents of the coffins, which were then buried in due form. They procured a key of the dead-house, and provided themselves with turn-screws, hammers, wrenching-irons, and everything likely to be wanted. The nurses and undertakers were allowed to take the ordinary course of laying out the subject and securing the coffin. Funerals were ordered generally for five o'clock, and during the hour or two preceding that time they used to steal into the dead-house, remove any of the corpse they wanted, even the whole body at times, and then made all fast and in the same order as before. Thus no suspicion was excited and the danger of an examination of the coffin rendered less probable. So eagerly was this pursued that there was scarcely a subject, unless immediately removed by the friends, which did not afford the students a demonstration, either of brain, or thorax, or abdomen, or the anatomy of an operation. In order to keep their secret of having a false key, they borrowed the real one occasionally from the old woman who kept the gate, and silenced her with a shilling now and then. Mr. Bowles was always their demonstrator; but as he was not a surgeon of the house, and as, moreover, there existed at that time a jealousy of him, it was necessary to smuggle him privately into the dead-house. This miserable place, which was in fact a mere coal-hole, lighted by a foot-square iron grating, was under ground, and had an opening into Lower

Maudlin Street. There the students spent hours in the ardent pursuit of anatomical knowledge, for there happened at that time to be a set of students who lost few opportunities for instruction.

In 1769 a complaint was made against the whole body of students at the Infirmary for removing the corpse from a coffin and substituting for burial a quantity of sand and wood. Being summoned to attend the Committee, and proving refractory, they were allowed a week to consider of it, being informed that if in that time due concessions were not made, a general Board would be summoned to settle the affair. The young gentlemen had to cry *peccavimus*, and an order was made that the key of the dead-house should always be in the custody of the apothecary.

One of the curious stories told by Richard Smith on this subject is the following:—

About the year 1750 a notorious vagabond, called Long Jack, having destroyed himself, a verdict of *felo-de-se* was pronounced against him. He was, in consequence, buried at the cross-roads leading to Kingswood. Mr. Abraham Ludlow formed with Mr. Page a scheme for the removal of the body, and Richard Smith accompanied them. A servant was behind leading a horse, with the resurrection implements. The subject was safely removed, and when the hole was filled up, the object of the expedition was fastened in a sack and laid across the horse. By this time, however, it was so late that the great portal of Castle Gate was closed, and no one could come through without leave of the porter.

This induced them to attempt driving the horse through the side avenue, intended only for foot passengers. In effecting this the body fell to the ground, and the porter, hearing a noise, came with his lantern, and was not a little alarmed to see the legs of a man at the mouth of the sack. He was, however, persuaded to hold his tongue, and the cavalcade reached Mr. Ludlow's house in safety. The body was placed upon a table in the back parlour, and the parties retired to rest themselves after their labours. But they had forgotten to lock the door, and in the morning when the servant girl opened the window she perceived the body of Long Jack, whom she very well knew. Horror-struck at the frightful appearance of a miserable-looking wretch with his throat cut, she ran screaming into the street, and Mr. Ludlow, awakened by the noise, had but just time to prevent the intrusion of the bystanders into his house. A buzz soon spread through the parish, and by evening it was so generally known, that some alarm was excited by the threats of some fellows to come in to know the truth. Mr. Ludlow, in consequence, resolved on replacing the body with all possible expedition. In the night, therefore, they contrived to deposit Long Jack at the place from whence they had carried him away. It was fortunate for them that they did so, for the next day a mob of fellows went to the spot to see if he were actually there or not, and vowed vengeance against all concerned in his removal in case they should not find him. A few strokes of the pickaxe having removed all their doubts as to his being there, they soon dispersed, and except an occasional joke from his professional brethren, Mr. Ludlow heard no more of the matter.

The following anecdote was related to Richard Smith by Mr. Godfrey Lowe. It occurred in 1761:—

A Kingswood lad died in the Infirmary of fractured skull, and Mr. Castleman, who was surgeon to the institution, had secured the specimen before the body was delivered in a coffin to the friends for interment. In the middle of the night there was a violent knocking at the door of the Infirmary, and the apothecary, who was known by no other name than "Neddy Bridges," thrusting his head and night-cap out of window, half asleep, yawned out, "What d'ye want?" "Want!" said a hoarse rough voice, "want! damn thee! why I da want my zun's head, and I 'll ha' en too, or else I 'll ha' thine!" Bridges endeavoured in vain to pacify him, and make him come on the morrow, but the fellow became outrageous, and continued to vociferate, "Gee I my zun's head; gee I my zun's head, or else I 'll zend a stwoan drough thine and pull the Furmery about thy ears!" Bridges, finding the matter becoming serious, was obliged to tell him that he must speak to Mr. Castleman, who lived hard by in Duke Street. Away went the man, and began to thunder away at that gentleman's, who, speedily throwing up the window, enquired, "Who 's there?" "Who 's there? Why, I be here!" "Well, who are you?" "Who be I? What! dostn't know me? Why, I be Jack's vather, and thee'st got his head; and if thee dostn't ge'en to me I 'll ha' thine, and there 's zomeut for year nest;" and with that he hurls a great stick up and knocks to pieces a pane of glass. Castleman, as he well might, feeling very much alarmed, told the man that it was at the Infirmary, and if he would step there he would come to him and get it. "Oh!" said the fellow, "thick 's the geam, is it? Measter Bridges zends I to thee, and thee zends I to he! I 'll tell thee what, if thee dostn't gee I my zun's head in vive minutes, I 'll smash every window thee'st got!" Seeing that all manœuvring was useless, Mr. Castleman went to his surgery, and wrapping the cranium in a towel, with no very pleasant feelings unbolted the street door, and delivered it into the hands of the collier, who had on the instant thrust himself into the hall. The fellow, that he might not be deceived, deliberately unfolded the cloth, and having exposed the countenance, said, "Aye, aye, thick 's Jack, and till's well vor thee that thee'st gid en to me, for if thee hadsn't, we 'd a come and had thy house down to-morrow." Mr. Castleman, half-naked, shivering with the cold, and extremely anxious to get his guest outside the door, civilly wished him a good-night. "Damn thy good-night!" said the collier: "what bizness had'st thee to cut poor Jack's head off? I got a despurt good mind now to gee thee a dowse in the chops, and then thee't know better another time;" at the same moment he grinned horribly a ghastly smile, and shook a tremendous fist at him. Mr. Castleman was too prudent to come between such a dragon and his wrath, and with as much composure as he could muster, begged his excuse for the trouble he had given him, and hoped that they should part friends. "Vrend, indeed!" said the fellow, "damn sitch a vrend as thee, or any o' the like o' thee!" and then, clapping the bundle with his "zun's head" under his arm, he stalked into the court, and went off grumbling to Kingswood, leaving Mr. Castleman to receive the congratulations of his wife, who was trembling at the head of the stairs, at his being delivered from so troublesome a customer.

Another curious story about the Infirmary, related by Richard Smith, is the following:—

One of the Infirmary surgeons, named Mr. John Ford, was in the habit of lounging in a poulterer's shop where there was a remarkably pretty girl. A passer-by remarked "how improper it was for a man of his rank and years to be dangling after a girl." "Pooh!" said another, "that is not his errand; why he goes there about the used poultices, they are sold to the poulterers by the Infirmary." This getting about, no one would buy poultry, and the shop was almost ruined. At length the matter was carried to such a height that the following affidavit appeared in the public newspapers (see *Pine's Gazette*, January, 1773):—

"Whereas it hath been currently reported in and about the city that the poulterers residing here have frequently purchased or received from the Infirmary divers quantities of poultices there made use of, for the purpose of feeding their poultry; whereby a great number of dealers and others have been led to think it hath been their general practice, and have declined further dealings with them. We, whose names are hereunder written, in justice to ourselves and families, and to clear as much as lies in our power our character and reputation from such a foul and infamous charge, think it proper to declare that we nor either of us, did ever, by ourselves servants or others, purchase or receive from the Infirmary, or any other place whatever, any poultice or other unwholesome thing, for the purpose of feeding or fattening our poultry. But it had been and shall continue to be our practice to feed them with the very best barley, barley-meal and other wholesome corn food, and with milk and clean water; and we hereby call on the author of this report and the propagators thereof to prove the contrary, by themselves or any other person.

| | |
|---|---|
| Sworn at the city of Bristol, | Signed |
| the 12th day of Jan., 1773, | CHRISTOPHER KEMPSTER, |
| before me | WILLIAM PRITCHARD, |
| NATHANIEL FRY, | + The Mark of |
| *Mayor.* | MARTHA JONES." |

Time would fail me to quote at any considerable length from the entertaining reminiscences and stories recorded by Richard Smith in these volumes. They bring vividly before us the everyday life of members of our profession in Bristol in the last century. They have been recorded with extraordinary diligence, and the mere labour of writing and arranging the memoirs must have been no light matter. It is to be regretted that they are not more accessible to members of this Society: perhaps some day we may be able to have copies made for the Bristol Medical Library; but at present they are jealously guarded by the Committee of the Infirmary, who were kind enough, however, to consent to my application to be allowed to read them and make extracts from them.

